# Obacunone reduces inflammatory signalling and tumour occurrence in mice with chronic inflammation-induced colorectal cancer

**DOI:** 10.1080/13880209.2020.1812673

**Published:** 2020-12

**Authors:** Xiaoping Luo, Zhilun Yu, Bei Yue, Junyu Ren, Jing Zhang, Sridhar Mani, Zhengtao Wang, Wei Dou

**Affiliations:** a Shanghai Key Laboratory of Formulated Chinese Medicines, Institute of Chinese Materia Medica, Shanghai University of Traditional Chinese Medicine (SHUTCM), Shanghai, China; b Departments of Medicine and Genetics, Albert Einstein College of Medicine, Bronx, NY, USA

**Keywords:** Inflammatory cytokines, colon cancer, colitis, cancer growth

## Abstract

**Context:**

Obacunone, a limonoid abundantly found in *Citrus* fruits, exhibits a variety of bioactivities.

**Objective:**

To investigate the effects of obacunone on a colorectal cancer (CRC) mouse model, and clarify its potential molecular mechanisms.

**Materials and methods:**

The male Balb/c mice were induced with azoxymethane and dextran sulfate sodium for 12 weeks. Obacunone (50 mg/kg) was administered via oral gavage three times every week until the end of the experiment. Disease indexes including body weight, spleen weight, bloody diarrhea, colon length, histopathological score, and tumor size were measured. The anti-proliferation activities of obacunone were analyzed by MTT or flow cytometry. The expression of protein and mRNA related to cell proliferation or inflammatory cytokines was determined by Western blot, q-PCR and IHC.

**Results:**

Obacunone significantly alleviated bloody diarrhea, colon shortening (7.35 ± 0.2128 vs. 8.275 ± 0.2169 cm), splenomegaly, histological score (9 ± 0.5774 vs. 6 ± 0.5774) and reduced tumor size (4.25 ± 0.6196 vs. 2 ± 0.5669). Meanwhile, the expression of protein and mRNA related to cell proliferation or inflammatory cytokines was remarkably decreased in tumor tissue. Obacunone inhibited the proliferation activities of colorectal cancer cells. Moreover, obacunone induced colorectal cancer cells G1 and G2 phases arrest, and suppressed the expression of cell cycle genes.

**Conclusions:**

Obacunone could alleviate CRC via inhibiting inflammatory response and tumor cells proliferation. The results may contribute to the effective utilization of obacunone or its derivatives in the treatment of human CRC.

## Introduction

Colorectal cancer (CRC) is the third most common cancer and the second leading cause of cancer-related death worldwide (Levin et al. 2008; Siegel et al. 2017). Accumulation of gene alterations and cell cycle dysregulation are widely accepted hallmarks of CRC morbidity but the aetiology of the disease remains elusive at present (Ewen 2000; Qin et al. 2019). Increasing evidence suggests that inflammatory bowel diseases (IBD), including ulcerative colitis and Crohn’s disease, promote the risk of developing carcinogenesis, such as CRC (Triantafillidis et al. 2009). For instance, a six times higher incidence of CRC in patients with IBD relative to the control population has been reported (Herrinton et al. 2012). Notably, colitis-associated CRC is characterized by chronic inflammation before the formation of aberrant crypt foci, polyps, adenomas, and carcinomas (Roncucci et al. 2008; Mariani et al. 2009; Yao et al. 2019). Chronic inflammation is one of the key underlying aetiologies of carcinogenesis, highlighting the importance of attenuating inflammation before tumour formation to suppress the initiation of tumorigenesis in the microenvironment.

Emerging research indicates that excessive levels of inflammatory cytokines and chemokines, including the interleukin family (IL-1, IL-6 and IL-12), tumour necrosis factor (TNF)-α, iNOS and COX-2, contribute to exacerbation of tissue damage and pathogenesis of CRC (Sakthivel and Guruvayoorappan 2013; Alpert et al. 2019; Lopes et al. 2019). Recent studies have shown that the beneficial effects of natural or synthetic chemicals on CRC are partly attributable to the suppression of pro-inflammatory cytokines (Rubila et al. 2016; Sakthivel and Guruvayoorappan 2018). 5-Aminosalicylic acid (ASA), a clinical IBD therapeutic drug, is documented to reduce the risk of CRC development in IBD patients. A number of reports suggest that non-steroidal anti-inflammatory drugs (NSAID) inhibit the development of CRC (Ota et al. 1999; Truong et al. 2019). Thus, anti-inflammatory therapy preventing the progression of inflammation to carcinogenesis may represent an effective approach to reduce the incidence of CRC.

According to the World Health Organisation (WHO) data, ~80% of the global population relies on herbal medicinal products as a complementary or alternative therapy, characterized by a wide range of sources, relatively low cost and long-term clinical practice (Ekor 2014). Obacunone, a natural limonoid, is mainly derived from *Citrus* and plants of the Rutaceae family. Multiple pharmacological effects of obacunone have been reported, including anti-inflammation (Gao et al. 2018), antitumor (Chidambara et al. 2011), antiproliferation (Kim et al. 2014), anti-obesity (Horiba et al. 2015) and antimalarial activities (Vikram et al. 2012). Tanaka et al. (2001) showed that an obacunone-containing diet reduced the incidence of colonic adenocarcinoma significantly in rats bearing colorectal tumours induced by azoxymethane (AOM). Moreover, Chidambara et al. (2011) demonstrated that obacunone inhibited cell viability of the human colorectal carcinoma cell line, SW480, via induction of apoptosis *in vitro*. In the current study, we focussed on the effects and underlying mechanisms of obacunone on chronic inflammation-associated colorectal carcinoma.

## Materials and methods

### Animals and chemicals

Balb/c mice (male, 6–8 weeks old, 20 ± 2 g) were purchased from the Shanghai Laboratory Animal Centre (Shanghai, China). All mice were housed in a specific pathogen-free environment at room temperature (25 ± 2 °C at a relative humidity of 55 ± 10%) under a 12 h light/dark cycle. Animals were acclimated for a week after arrival. All laboratory experiments were conducted according to the guidelines of the Institute of Animal Care and Use Committee of Shanghai University of Traditional Chinese Medicine using protocols approved by the Animal Care Ethics Committee of Shanghai University of Traditional Chinese Medicine (No. SZY201506001).

### Reagents and antibodies

Azoxymethane (AOM) was purchased from Sigma-Aldrich (St. Louis, MO) and DSS (molecular mass, 36–50 kDa) from MP Biochemical (Irvine, CA). 5-Bromo-2-deoxyuridine (BrdU, ab142567) was obtained from Abcam (Cambridge, MA) and obacunone (purity ≥ 98%) from Chengdu Biopurify Phytochemicals Ltd. (Chengdu, China).

Rabbit antibodies against iNOS (#13120), COX-2 (#12282), TNF-α (#3707), p-p65 (#3033), cyclin A2 (#4656) and cyclin E1 (#4136), P21(#2947) were obtained from Cell Signalling Technology (Danvers, MA). Mouse antibodies against p-IκBα (sc-8404) and β-actin (sc-47778) were purchased from Santa Cruz Biotechnology (Santa Cruz, CA) and the ELISA kit (IL-6, TNF-α) were bought from Nanjing Jiancheng Bioengineering Institute (Nanjing, China).

### AOM/DSS-induced colitis-associated colorectal cancer and design of treatment drugs

**Figure 1.**
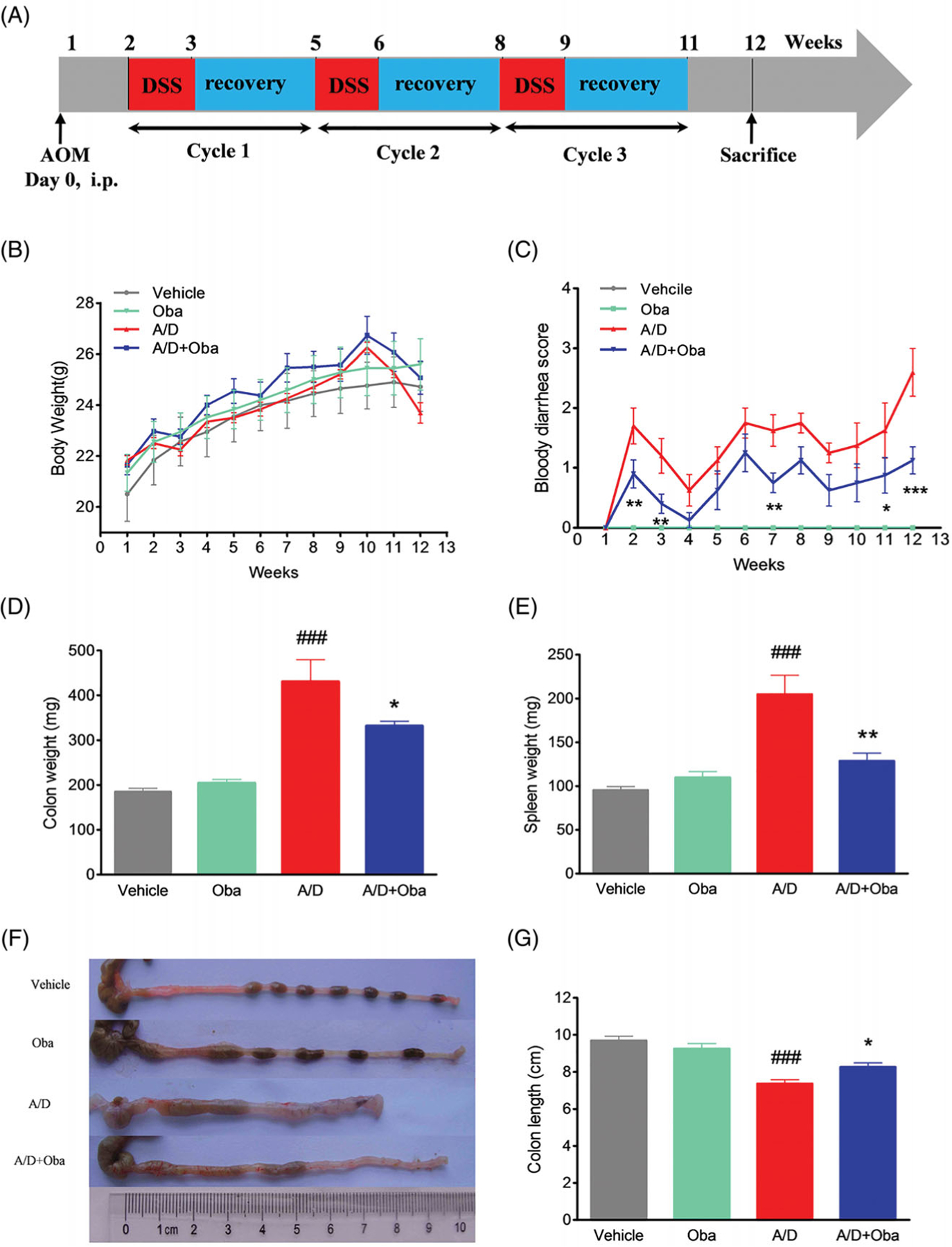
Obacunone alleviated AOM/DSS-induced colitis in CRC mice. Experimental schematic overview of the procedures used for generating the AOM/DSS mouse model (A). After intraperitoneal injection with AOM (10 mg/kg), 2.5% DSS was added to drinking water for 7 days, followed by administration of tap water. The cycle was repeated another twice. All mice were sacrificed after 12 weeks of AOM injection. Changes in body weights of individual mice (B) and scores of bloody diarrhoea (C) were measured three times a week during the experiment. After sacrifice, the colon and spleen weights were measured (D, E). Colon lengths of mice were determined (F, G). Values are expressed as means ± SD (*n* ≥ 6). vs Vehicle group, ###*p* < 0.001; vs A/D group, *p < 0.05; **p < 0.01; ***p < 0.001.

All mice were randomly divided into the vehicle (Vehicle, *n =* 6), obacunone control (Oba, *n =* 6), AOM/DSS (A/D, *n =* 8), and AOM/DSS obacunone (A/D + Oba, *n =* 8), respectively. The murine CRC model was established in Balb/c mice by the administration of AOM with repeated DSS exposure. The procedure was performed as specified for Figure 1(A). Briefly, mice were injected with AOM (10 mg/kg, i.p.) on experimental day 0. One week later, mice were given a course of 2.5% DSS in drinking water for one week followed by tap water for two weeks. These steps were repeated twice. Obacunone (50 mg/kg body weight) or an equal volume of 0.5% CMCNa (carboxymethyl cellulose sodium) was administered via oral gavage three times every week until the end of the experiment. After this period, blood was collected from mice under anaesthesia. The colon was removed, rinsed with ice-cold PBS (pH 7.4), and opened longitudinally. The colon length was measured and weights of colon and spleen were obtained. The extent of dysplasia and carcinoma in the colon was evaluated macroscopically and recorded. Next, colon sections were cut into ~1.5 cm slices for fixing into 4% paraformaldehyde buffer (pH 7.4). The remainder of the colon was fast-frozen in liquid nitrogen and stored at −80 °C for subsequent mRNA and protein analyses.

### Histopathologic (HE) and immunohistochemistry (IHC) analyses

Histological alterations were examined via standard haematoxylin-eosin (HE) staining. For histopathological examination, the colon was fixed in paraformaldehyde, embedded in paraffin, cut into serial sections (5 µm), and stained with haematoxylin and eosin. The severity of colitis was evaluated by two blinded pathologists. Histological scores were calculated as the total score of inflammatory cell infiltration (0–3) and tissue damage (0–3), as described previously (Wallace et al. 2010; Dou et al. 2013).

Protein levels of COX-2 and PCNA in colonic tissues were measured using the respective polyclonal antibodies. Slides were subjected to antigen retrieval, washed with 1 × PBST (8.36 mM NaH_2_PO_4_, 0.137 mM KCl, 1.47 mM KH_2_PO_4_, 0.1% Tween-20, pH 7.4) and blocked with 5% BSA (albumin from bovine serum) in 1× PBST. Sections were incubated with primary antibody or normal IgG, and after three washes, incubated with horseradish peroxidase-conjugated secondary antibody. Finally, tissue sections were incubated with DAB (diaminobenzidine substrate), washed and mounted.

### *Cell proliferation assay* in vivo *(BrdU)*

Half of the mice in each group were injected with 100 mg/kg body wt (i.p.) BrdU and sacrificed 3 h later. At the 3 h time-point, mice were euthanized, followed by colon removal. To determine BrdU incorporation, sections were subjected to *in situ* immunohistochemical staining with an anti-BrdU antibody.

### Measurement of IL-6 and TNF-α levels in colonic tissue

Colon sections were washed and homogenized in cold phosphate-buffered saline (PBS) with a high-flux tissue homogenizer. After centrifugation at 12,000 *g* for 15 min, supernatant fractions were collected for determination of IL-6 and TNF-α levels with ELISA kits following the manufacturer’s protocols. The results were presented as ng/L for each sample.

### Cell culture

Caco2, SW480, HCT-116, HT-29 cells derived from human colon cancer were obtained from Shanghai Institutes for Biological Sciences (Shanghai, China) and cultured at 37 °C in a humidified atmosphere containing 5% CO_2_.

### Cell viability assay

Cell viability was detected with the 3-[4,5-dimethylthiazol-2-yl]-2,5-diphenyl tetrazolium bromide (MTT) assay. Human colon cancer cells were seeded in 96-well plates at a density of 20,000 cells/well and incubated overnight. Each trial was conducted in triplicate for all obacunone doses (0–200 µM) for 48 h. The old culture medium was removed and 100 µL fresh medium containing 10% MTT (5 mg/mL) added to each well and incubated at 37 °C for 4 h, followed by removal of culture medium. Tetrazole Red Formazan (100 µL/well) was dissolved, fully shaken, and incubated at 37 °C for 1 h. Absorbance was detected using a Universal Microplate Reader (Thermo Fisher, Waltham, MA) at 570 nm, and results were presented as means ± SEM.

### Western blot (WB) analysis

Colon tissues or cancer cells were homogenized and completely lysed to extract total proteins in cold RIPA containing protease inhibitors. Homogenates were centrifuged at 12,000 *g* for 15 min at 4 °C and protein concentrations in the supernatants detected with the BCA kit. An aliquot (30 mg) of protein for each sample was subjected to 10 or 12% SDS-PAGE, followed by transfer to polyvinylidene fluoride membrane (PVDF). PVDF was blocked in 5% BSA for 2 h and incubated overnight with primary antibody and subsequently the associated secondary antibody for 1 h. Blots were visualized using the enhanced chemiluminescence detection kit and images assessed using an Automatic Chemiluminescence Image Analysis System (Tanon 5200). Results were calculated as density ratio and quantified by normalization to the intensity of β-actin control.

### Real-time quantitative (q) PCR analysis

qPCR was conducted following the manufacturer’s instructions. Total RNA was extracted from colon samples and cultured cells using TRIzol reagent. The RNA concentration was determined by the nano spectrophotometer. RNA (1.5 µg) from each sample was subjected to reverse transcription using the PrimeScript Reverse Transcriptase kit (TaKaRa, NO. RR036A) and qPCR analysis performed according to the protocol of TaKaRa SYBR Green Master Mix Kit (TaKaRa, Tokyo, Japan). Primer sequences are presented in Table 1. Gene expression was analyzed via comparative Ct with β-actin as the calibrator and normalized to that of vehicle-treated cells. The fold changes in expression of individual genes were analyzed using the 2^−ΔΔCt^ method.

### Cell cycle assay

After treatment with obacunone, Caco2 cells were washed, digested with 0.25% trypsin and collected by centrifugation. Cells were washed twice with 1 × DPBS and resuspended in cold 70% ethanol at −20°C for at least one day, collected, and subjected to two further washes. Next, cells were stained with 5 µL of 20 µg/mL propidium iodide (PI) for 15 min and assessed via flow cytometry on a BD instrument (San Jose, CA) in 1 h.

### Statistical analysis

All the experiments were performed independently at least three times. Significance between groups was evaluated by one-way analysis of variance (ANOVA) using GraphPad Prism 7 software (GraphPad Software, La Jolla, CA).

## Results

### Obacunone inhibits intestinal tumour cell proliferation

**Figure 2.**
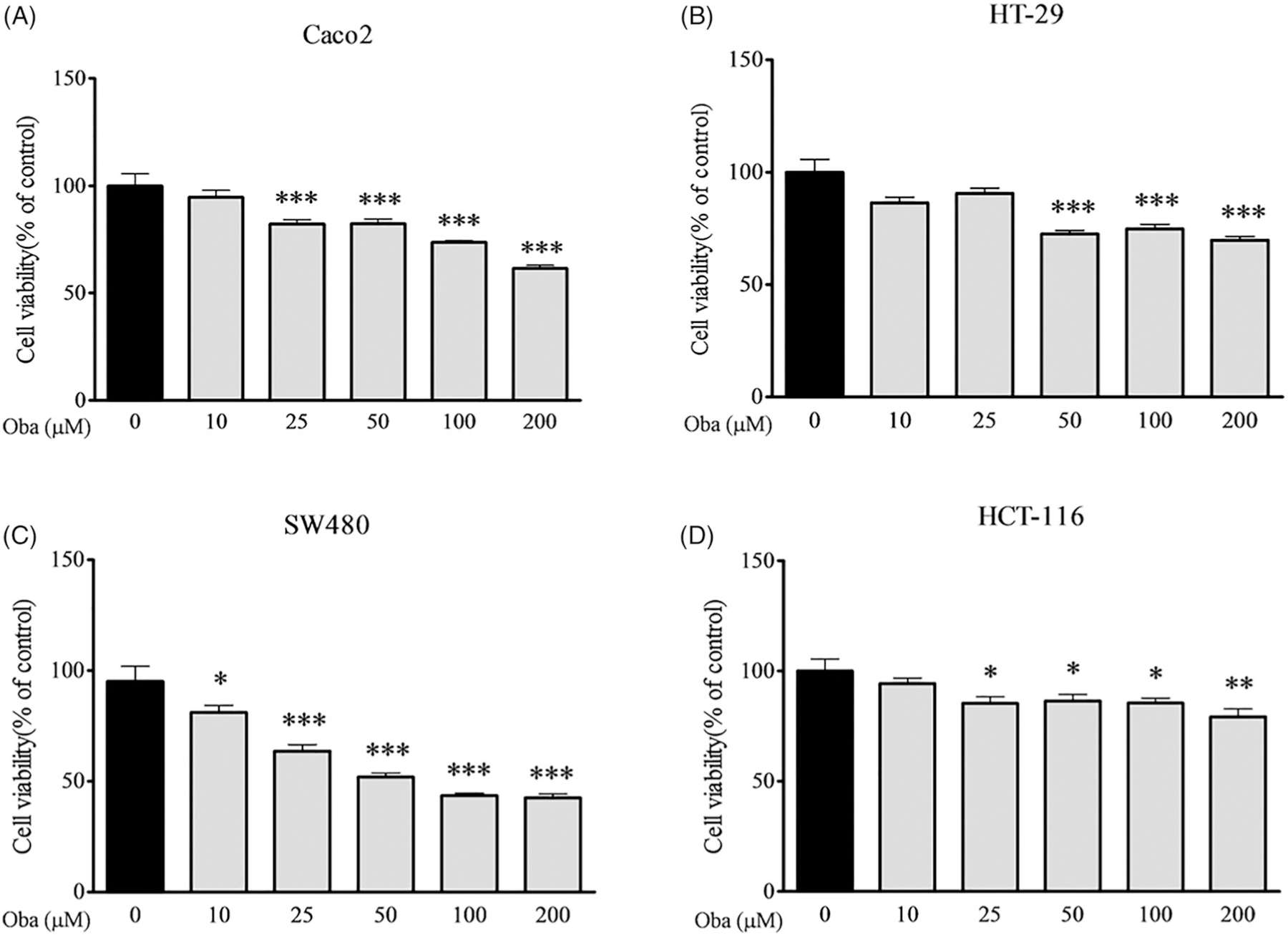
Obacunone inhibited the viability of human colon cancer cells. Human colon cancer cells were treated with different concentrations of obacunone for 48 h, and subjected to the MTT assay for detection of cell viability. (A) Caco2 cells, (B) HT-29 cells, (C) SW480 cells, (D) HCT-116 cells. Values are expressed as means ± SD (*n* = 4). vs control group, *p < 0.05; **p < 0.01; ***p < 0.001.

Since cancer cells are essential tools for preclinical testing of potent anticancer bioactivities *in vitro*, we detected the viability of several human colorectal carcinoma cell lines following obacunone treatment using the MTT assay. Obacunone suppressed the viability of Caco2, HT-29, SW480 and HCT-116 cells after 48 h exposure in a concentration-dependent manner (Figure 2(A–D)), indicating potent antiproliferative effects on specific colorectal cancer cell lines.

### Obacunone suppresses colitis-associated colorectal tumorigenesis in the AOM/DSS mouse model

**Figure 3.**
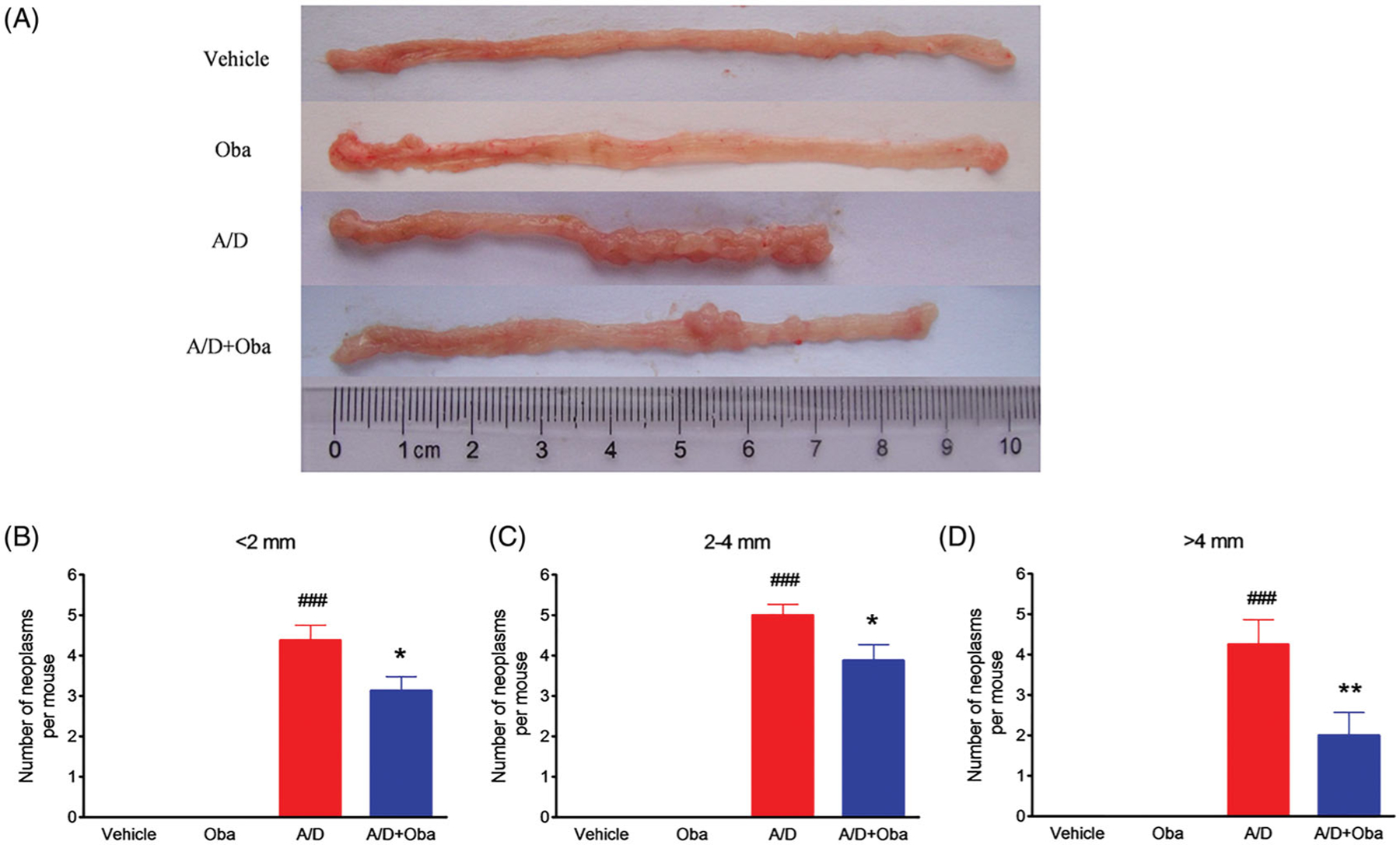
Obacunone prevented tumour growth in a CRC model. Representative views of mouse colons (A). Statistical data on tumour number and tumour size in mice (B–D). Values are expressed as means ± SD (*n* ≥ 6). vs Vehicle group, ###*p* < 0.001; vs A/D group, *p < 0.05; **p < 0.01.

We further assessed the antitumor effects of obacunone on the AOM/DSS murine model. Colitis-associated colorectal cancer was induced in mice by intraperitoneal injection of AOM, followed by three cycles of DSS administration. The study scheme was summarized in Figure 1(A). As expected, body-weight loss was evident following DSS treatment but recovered within the period of administration of normal tap water. Overall, no significant difference in body weight loss between the model and obacunone treatment groups were observed (Figure 1(B)). Next, the severity of diarrhoea and bloody stool was assessed via sequential monitoring. As shown in Figure 1(C), obacunone treatment led to a significant decrease in the severity of bloody diarrhoea. DSS administration induced an increase in colon and spleen weights, which was suppressed by obacunone (Figure 1(D,E)). Since colon shortening presents a marker for the severity of intestinal inflammation, we performed macroscopic examination of colon length. The colon lengths of mice in the AOM/DSS model group were remarkably decreased, compared to those of control mice. Notably, obacunone administration prevented shortening of colon length caused by AOM/DSS treatment (Figure 1(F,G)). After the colon was opened longitudinally, numerous neoplasms and nodular tumours in the middle and distal regions were observed in the AOM/DSS model group. Treatment with obacunone induced a significant decrease in the incidence and size of tumours (Figure 3(A–D)).

**Figure 4.**
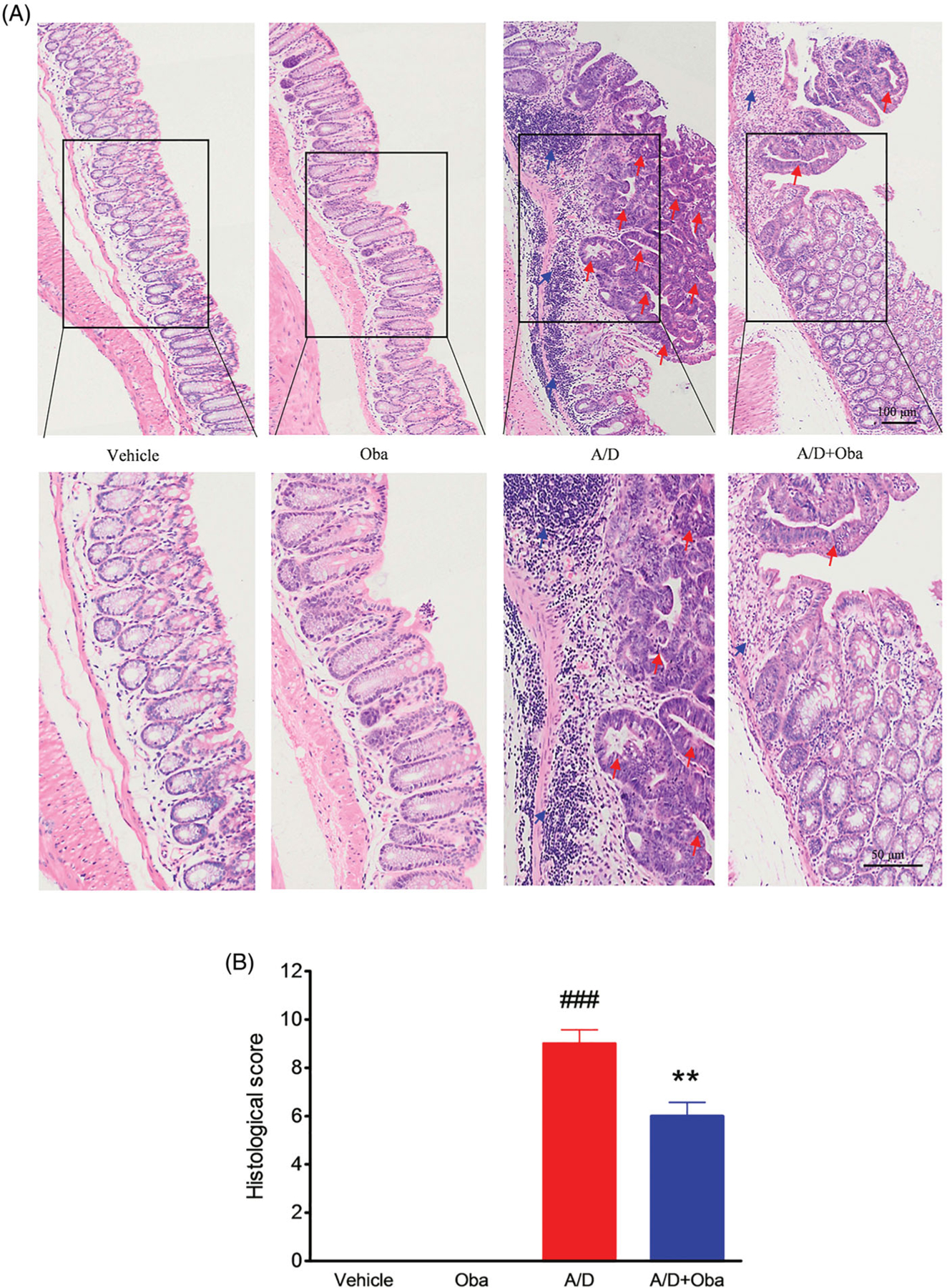
Obacunone reduced histologic pathological changes in CRC. H&E staining of the colon from different groups (A). Macroscopic inflammation scores of H&E staining (B). The arrows indicate the neoplastic areas or inflammatory areas. Values are presented as means ± SD (*n ≥* 6). vs Vehicle group, ###*p* < 0.001; vs A/D group, **p < 0.01.

Next, the histological analysis of colon sections was performed. We observed no inflammation or colonic neoplasms in the intestines of both control and obacunone treatment groups. However, the AOM/DSS model group displayed 100% incidence of mucosal inflammation, aberrant crypt foci, adenoma, dysplasia or colonic tubular neoplasms, accompanied by excessive epithelial structure loss and extensive neutrophil cell infiltration. Mice treated with obacunone exhibited less colonic epithelial denudation, lower inflammatory reaction, smaller and fewer adenomas and dysplasia in the colon, compared with the AOM/DSS model group (Figure 4(A)). Correspondingly, the histological score of the obacunone treatment group was lower than that of AOM/DSS model group (Figure 4(B)).

Furthermore, no significant differences in the disease hallmarks between vehicle and obacunone control groups were evident, supporting the relative safety of obacunone administration. Our collective data clearly demonstrate that obacunone inhibits colon tumorigenesis in the AOM/DSS-induced colorectal cancer mouse model.

### Obacunone suppresses pro-inflammatory mediator production

**Figure 5.**
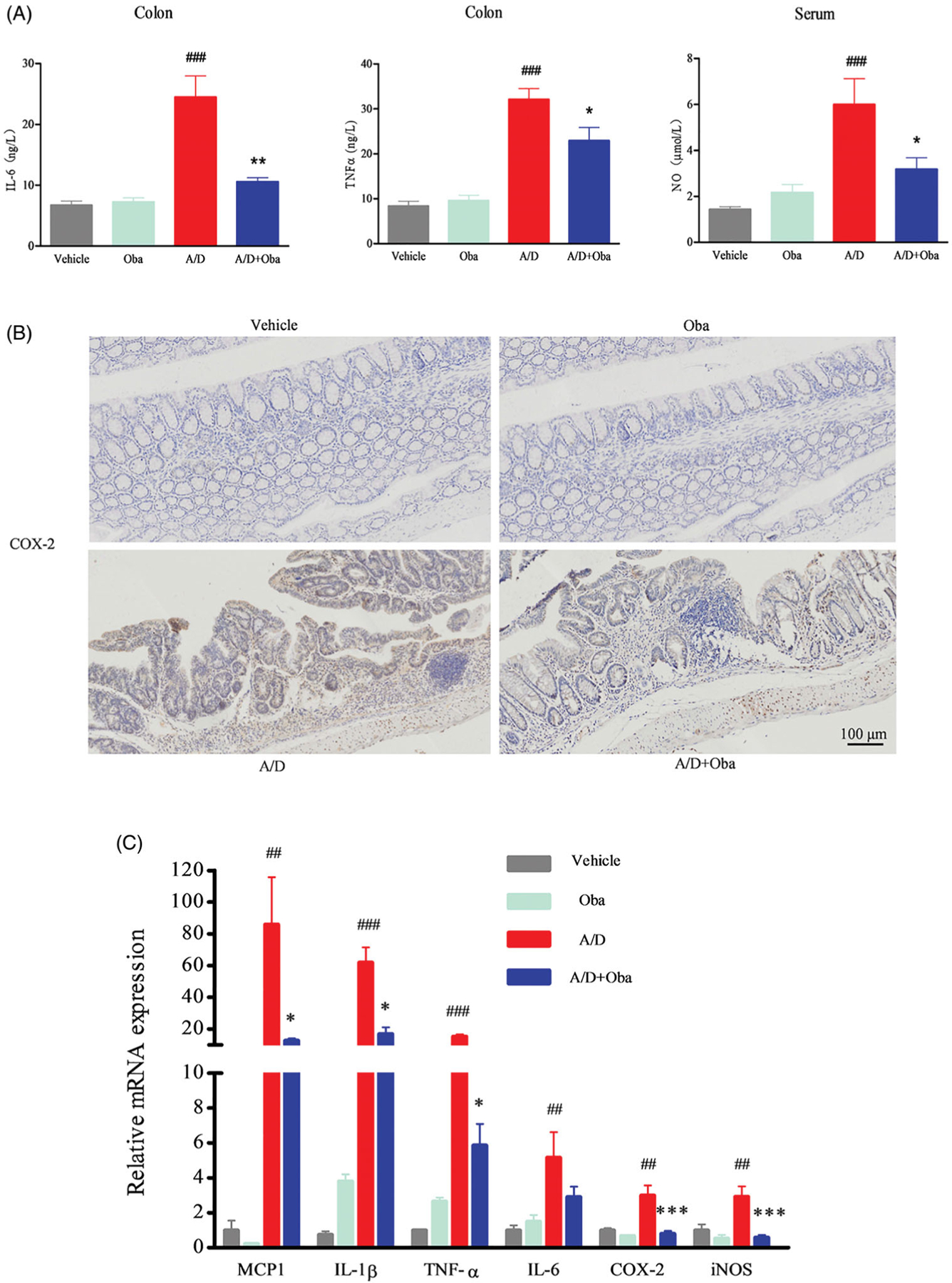
Obacunone inhibited the expression of inflammatory cytokines in CRC. ELISA evaluation of inflammatory cytokine (IL-6, TNF-α, NO) levels in different groups (A). IHC staining for assesment of the COX-2 level (B). qPCR analysis of relative mRNA expression of inflammatory cytokines (C). Values are presented as mean ± SD (*n* ≥ 6). vs Vehicle group, ##*p* < 0.01, ###*p* < 0.001; vs A/D group, *p < 0.05, **p < 0.01, ***p < 0.001.

**Figure 6.**
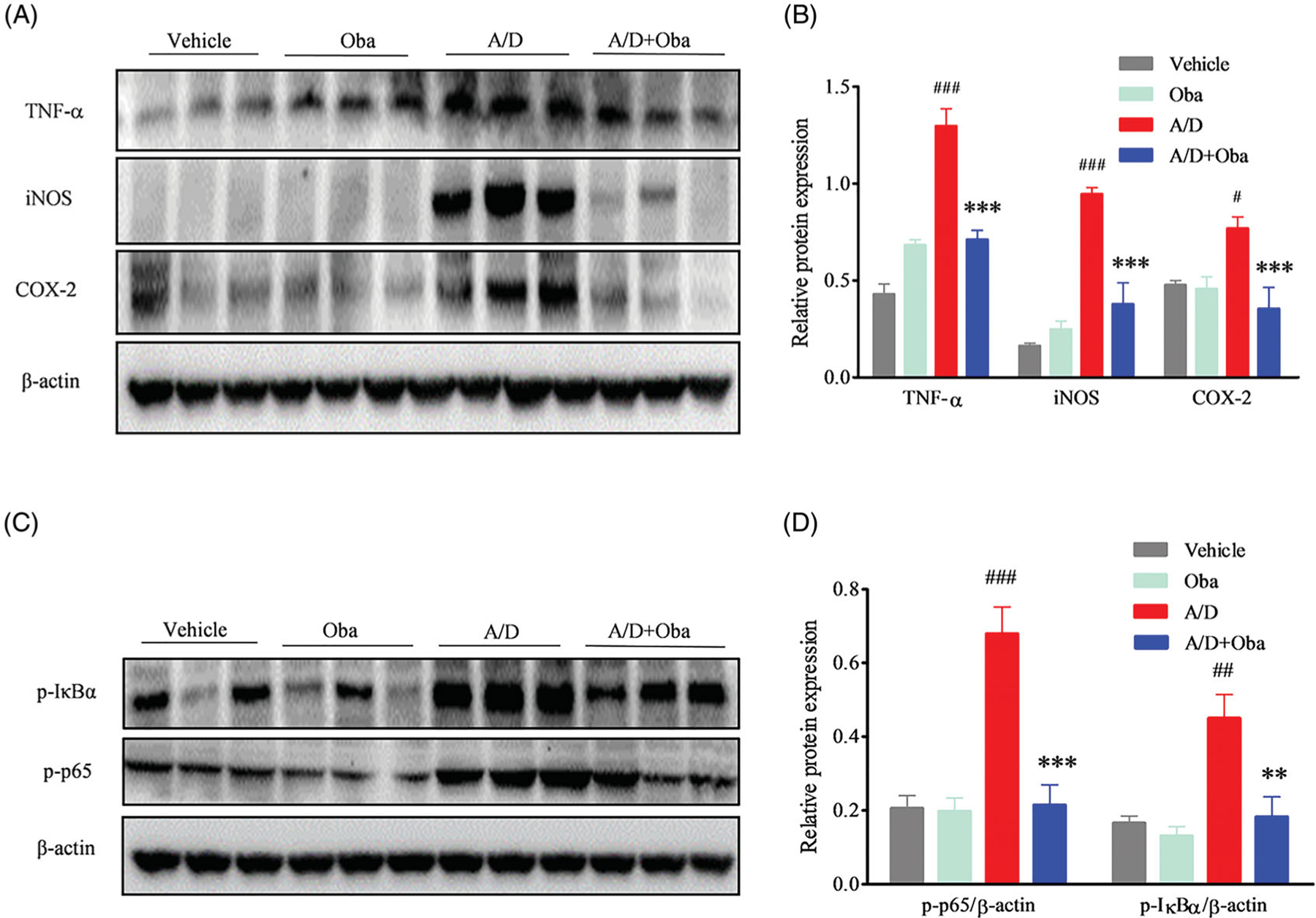
Obacunone suppressed the protein levels of pro-inflammatory cytokines and phosphorylation of the NF-κB complex. Protein levels of TNF-α, iNOS, COX-2 (A) and NF-κB complex (C) in colon tissue were measured via WB. Relative densities of these proteins were calculated (B, D). Values are presented as means ± SD (*n* = 3). vs Vehicle group, #*p* < 0.05; ###*p* < 0.001; vs A/D group, **p < 0.01; ***p < 0.001.

Chronic inflammation is one of the major underlying causes of tumour initiation and development. Cytokines and chemokines are known to play a pivotal role in promotion of inflammation. Accordingly, we assessed the production of pro-inflammatory mediators in AOM/DSS-treated mice via ELISA. As shown in Figure 5(A), obacunone reversed the increase in IL-6, TNF-α and NO production after AOM/DSS exposure. No differences in the production of IL-6, TNF-α and NO were observed between the obacunone treatment and non-treatment groups. COX-2 expression in colonic mucosa was additionally determined via immunohistochemistry (IHC). Compared with vehicle mice, the AOM/DSS model group exhibited significant induction of COX-2 in colon tissue, which was restricted by obacunone (Figure 5(B)). We further performed qPCR analysis of the expression levels of pro-inflammatory mediators in colonic tissue. As shown in Figure 5(C), treatment with AOM/DSS caused a marked increase in MCP-1, IL-1β, TNF-α, IL-6, COX-2 and iNOS mRNA levels in the colonic mucosa, which were downregulated following obacunone treatment. Protein expression of TNF-α, iNOS and COX-2 in colonic tissue was additionally determined via immunoblot analysis. As expected, levels of these proteins were remarkably elevated in the inflamed colon of AOM/DSS mice. Obacunone exerted a significant inhibitory effect on the expression of these pro-inflammatory mediators, consistent with ELISA, IHC and qPCR findings (Figure 6(A)).

### Obacunone inhibits NF-κB activation

NF-κB is the major nuclear transcription factor mediating the expression of pro-inflammatory mediators. IκB is an inhibitor of NF-κB p65 and its phosphorylation is a prerequisite for activation of NF-κB p65. Accordingly, we evaluated the phosphorylation levels of IκBa and p65 in colonic tissue. As shown in Figure 6(B), levels of phosphorylated IκBa and p65 in the colon were increased by AOM/DSS, compared with the vehicle group. This abnormal activation of phosphorylation was suppressed by obacunone.

### Obacunone reduces tumour cell proliferation in colonic tissue

**Figure 7.**
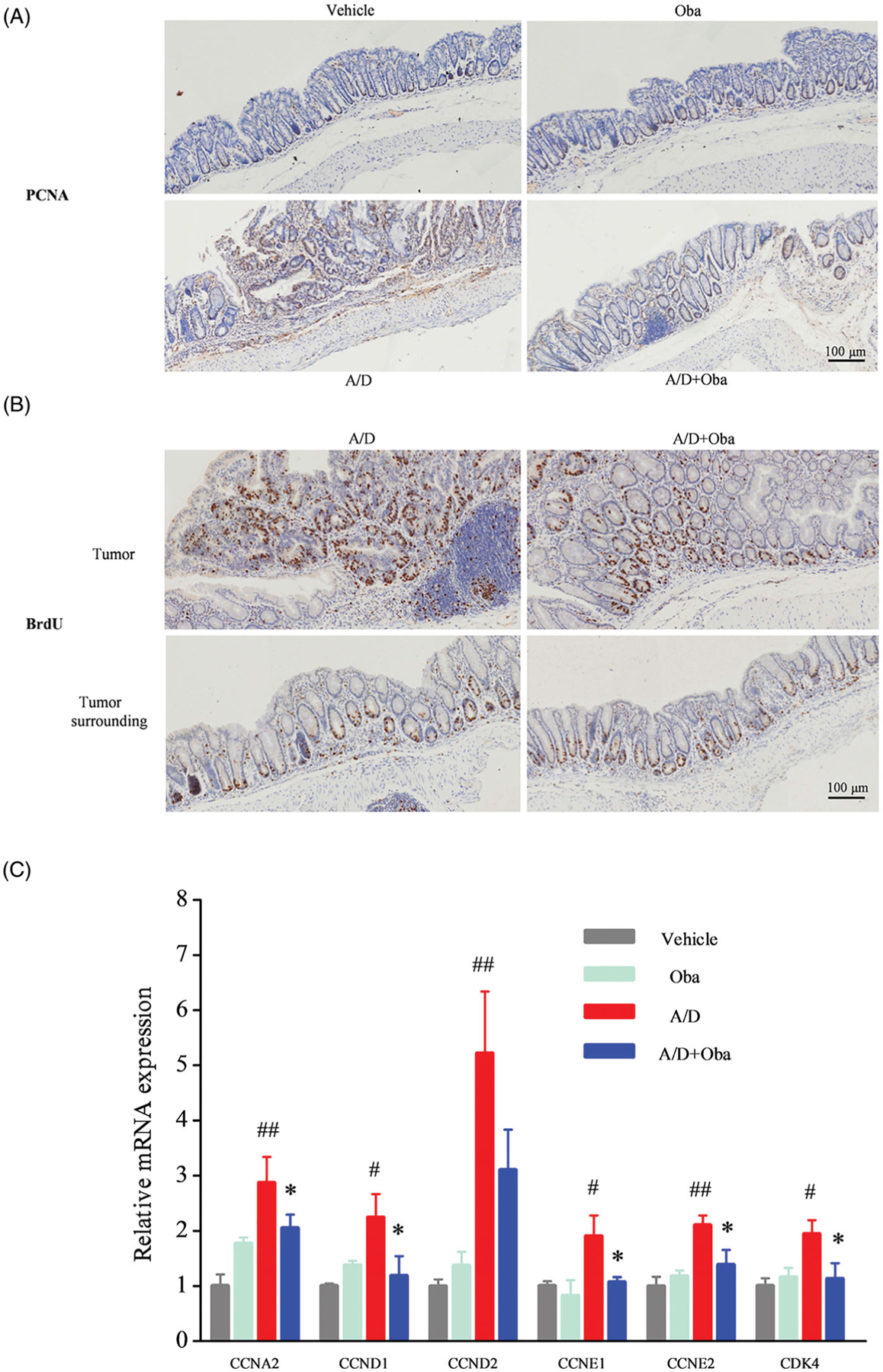
Obacunone inhibited the cell proliferation *in vivo*. PCNA (A) and BrdU (B) levels in different groups were detected via IHC staining. Relative mRNA expression of the cell cycle-associated genes in colonic tissue (C). Values are presented as means ± SD (*n =* 4). vs Vehicle group, #*p* < 0.05; ##*p* < 0.01; vs A/D group, *p < 0.05.

Tumorigenesis is initiated and expanded by uncontrolled cell proliferation (Sonnenschein and Soto 2013). Proliferating cell nuclear antigen (PCNA) is generally overexpressed in tumour cells and commonly used as an index of cell proliferation (Stoimenov and Helleday 2009; Muller et al. 2013). IHC was employed to detect the expression of PCNA in the colonic mucosa, which revealed increased expression in dysplastic areas of the AOM/DSS model group (Figure 7(A)). Following the administration of obacunone, the expression of PCNA in ulcers was reduced. 5-Bromo-2′-deoxyuridine (BrdU) incorporation is generally used to study the proliferative status of cancer cells (Matatall et al. 2018). Accordingly, we assessed the effect of obacunone on carcinogenesis via short-term injection of BrdU followed by immunostaining to evaluate BrdU-labeled intestinal epithelial cells. As shown in Figure 7(B), AOM/DSS treatment caused a marked increase in the number of proliferating cells while treatment with obacunone led to fewer proliferating cells in inflamed mucosa. Consistent with immunostaining results, mRNA levels of cell proliferation-related genes (*CCNA2*, *CCND1*, *CCND2*, *CCNE1*, *CCNE2* and *CDK4*) were markedly increased in the inflamed colons of AOM/DSS mice and reduced in the presence of obacunone (Figure 7(C)).

### Obacunone reduces colon tumour cell proliferation

**Figure 8.**
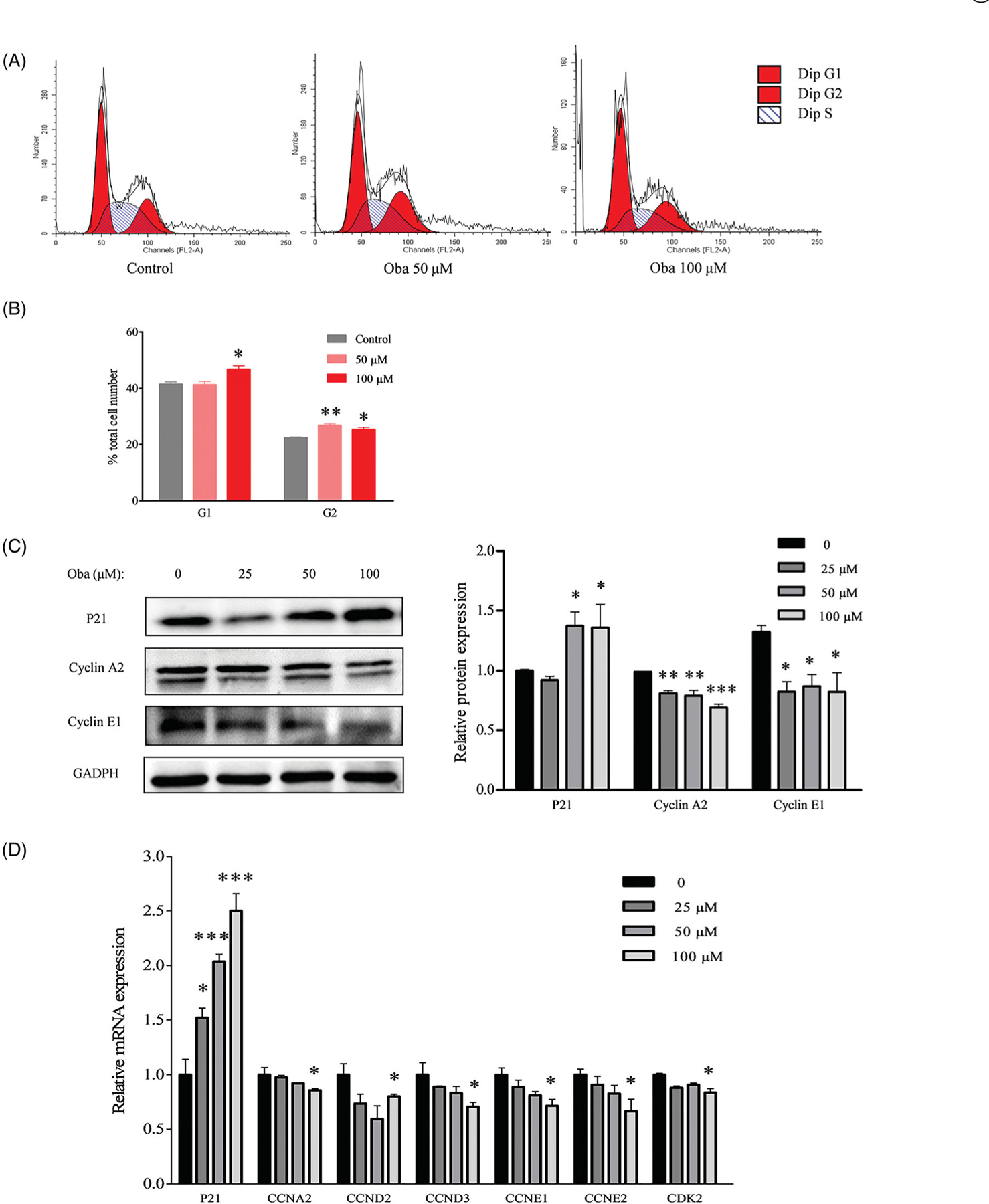
Obacunone suppressed mRNA and protein levels of cell cycle-associated genes *in vitro*. Flow cytometry was used to analyze the cell cycle of Caco2 cells treated with or without obacunone (A). Relative cell numbers in the G1 and G2 phases (B). Levels of proteins related to the cell cycle in Caco2 cells were determined via WB and their relative densities calculated (C). Relative levels of mRNAs associated with the cell cycle in Caco2 cells treated with/without obacunone were assessed via qPCR (D). Values are presented as means ± SD (*n* = 3). vs control group, *p < 0.05, **p < 0.01, ***p < 0.001.

The cell cycle is crucial in the regulation of cellular proliferation and a central hallmark of oncogenesis (Sonnenschein and Soto 2013). To further evaluate the inhibitory effects of obacunone on colorectal carcinoma *in vitro*, flow cytometry was employed to evaluate the cell cycle of human colorectal carcinoma (Caco2) cells. Compared with control cells, Caco2 cells were arrested at the G1 and G2 phases after exposure to 50 µM or 100 µM obacunone for 48 h (Figure 8(A,B)), consistent with the repressive effects of obacunone on Caco2 cell viability (Figure 2(A)).

The cell cycle is precisely controlled by specific proteins, including P21, cyclin A2, and cyclin E1. P21, a tumour suppressor gene, is involved in the regulation of cell proliferation by inhibiting the cyclin-dependent kinase (CDK) complex (Karimian et al. 2016). Cyclin A plays a role in the rate-limiting step for entry into mitosis and its overexpression accelerates the G1 to S transition causing DNA replication (Furuno et al. 1999). Accumulation of cyclin E at the transitional period of G1-S accelerates cells entry into the S phase (Lundberg and Weinberg 1998; Ewen 2000). We, therefore, performed immunoblot analysis of the protein levels of P21, cyclin A2, and cyclin E1 in Caco2 cells. Our results showed a significant increase in the P21 protein level after obacunone treatment (Figure 8(C)). Conversely, we observed a markedly decrease in cyclin A2 and cyclin E1 protein levels in Caco2 cells. Similarly, obacunone exerted prominently suppressive effects on mRNA levels of cell proliferation-related genes (*CCNA2*, *CCND2*, *CCND3*, *CCNE1*, *CCNE2*, *CDK2*, and *P21*; Figure 8(D)).

## Discussion

Together with the hereditary syndromes of familial adenomatous polyposis and hereditary nonpolyposis colorectal cancer, IBD is among the top three high-risk conditions for CRC. The murine CRC model induced by AOM with repeated exposure to DSS has been widely applied to elucidate the role of IBD in CRC during the past few decades (Sun et al. 2016; Bader et al. 2018; Lu et al. 2019). In this model, three cycles of DSS are applied to induce chronic inflammation, mimicking IBD. Induction of long-lasting inflammation provides a viable environment for normal to malignant transformation of epithelial cells (Elinav et al. 2013; Yao et al. 2019). Therefore, techniques that inhibit inflammatory progression and tumour initiation may achieve efficient prevention of CRC.

Obacunone, a natural bioactive compound abundantly found in citrus fruits, possesses potent anti-inflammatory and anti-tumour properties. Our previous research found that obacunone attenuated experimental colitis in mice through modulation of the gut microbiota, attenuation of TLR4/NF-κB signalling cascades, and restoration of intestinal epithelial barrier integrity (Luo et al. 2020). Data from the current study showed that obacunone inhibits the proliferation of several colon carcinoma cell lines (Caco2, SW480, HCT-116, HT-29). In addition, we used a chronic colitis-related CRC mouse model by injection of AOM followed by three-cycle DSS administration, with a view to elucidating the long-term effect of obacunone on CRC. Notably, obacunone attenuated the common characteristics of colitis, including diarrhoea, bloody stool, colon edoema, colon weight, shortening of colon length, spleen swelling, histological injury, neutrophil infiltration and lamina propria accumulation of inflammatory cells, clearly implying that obacunone effectively ameliorates AOM/DSS-induced colitis during the early stages of tumour initiation. Moreover, fewer dysplasia areas and adenomas were observed in AOM/DSS mice receiving obacunone treatment, leading to the suggestion that Obacunone suppresses tumour occurrence and proliferation in late AOM/DSS-induced CRC.

The mechanism underlying IBD-associated CRC is complex but carcinogenesis is widely accepted to be associated with the durational inflammatory microenvironment (Triantafillidis et al. 2009; Ullman and Itzkowitz 2011). Pro-inflammatory cytokines have been identified as major contributors to the inflammatory background during the initiation and development of IBD and CRC (Yao et al. 2019). Significantly enhanced levels of pro-inflammatory cytokines, such as IL-1β, IL-6, TNF-α, iNOS, COX-2, under CRC conditions are reported (Madka and Rao 2013; Sun et al. 2016). Consistent with earlier findings, protein and mRNA levels of pro-inflammatory genes were markedly increased in inflamed colonic tissues of the AOM/DSS group in our study. However, treatment with obacunone induced a significant decrease in levels of the genes examined. NF-κB, a major cellular transcriptional factor, promotes initiation and amplification of inflammation by regulating pro-inflammatory cytokine expression (Soleimani et al. 2020). Prolonged NF-κB activation accelerates progression from IBD to CRC, therefore facilitating tumour development of IBD-related CRC (Wang et al. 2019; Adachi et al. 2020). Accumulating evidence has demonstrated the phosphorylation of NF-κB in IBD and CRC patients (Li et al. 2019; Soleimani et al. 2020). In our experiments, the phosphorylation status of NF-κB was remarkably suppressed following obacunone administration. While the exact targets of obacunone remain to be established, the compound clearly reduced inflammation and inhibited tumour development by suppressing production of inflammatory cytokines and activation of the NF-κB pathway.

Cell proliferation is an important indicator of cancer (Wang et al. 2015). PCNA and BrdU are often overexpressed in tumour cells and commonly used as markers of cell proliferation. PCNA is the proliferative-associated protein controlling cell growth (Bravo et al. 1987). BrdU, a thymidine analog, readily incorporates into proliferating cells (Kee et al. 2002). Injection of BrdU into mice for 3 h before sacrifice allows incorporation into intestinal DNA in place of thymidine. A combination with anti-BrdU for IHC analysis indicates the status of cell proliferation *in situ*.

In our study, lower staining of PCNA and BrdU in colonic dysplastic areas was observed in the obacunone treatment group, compared with AOM/DSS group. Of note, no significance in staining of the adjacent normal tissue. This result suggests that obacunone exerts anti-proliferative effects on tumour cells, but not normal cells. *In vitro*, cell cycle detection was used to examine cell proliferation status. Flow cytometry analysis of human colon cancer samples disclosed that obacunone arrested the cell cycle process, leading to the accumulation of cells in the G1 and G2 phases. Similarly, protein and mRNA levels of cell cycle-associated genes were suppressed following obacunone treatment *in vivo* and *in vitro*. In view of the collective findings, we propose that the anti-proliferative effect of obacunone effectively contributes to suppression of tumour development in CRC.

## Conclusions

Obacunone significantly abrogated the severity of AOM/DSS-induced CRC in mice. Mechanistically, the beneficial effects of obacunone were attributable to suppression of tumour initiation through inhibitory effects on inflammation and preventing tumour development through suppression of cell proliferation. Our findings support the efficacy of obacunone as a potential therapeutic agent for IBD associated CRC in the future.

## Figures and Tables

**Table 1.** The list of genes sequence for RT-PCR.

Gene name	Forward sequence	Reverse sequence
*m MCP1*	ACCTGCTGCTACTCATTCAC	ATTCCTTCTTGGGGTCAG
*m IL-1β*	GCAACTGTTCCTGAACTCAACT	ATCTTTTGGGGTCCGTCAACT
*m TNF-α*	CTCTTCTCATTCCTGCTTGT	GTGGTTTGTGAGTGTGAGG
*m IL-6*	GCCTTCCCTACTTCACAA	ACAACTCTTTTCTCATTTCCAC
*m COX-2*	GAAGTCTTTGGTCTGGTGCCT	GCTCCTGCTTGAGTATGTCG
*m iNOS*	GTTCTCAGCCCAACAATACAAGA	GTGGACGGGTCGATGTCAC
*h Ccnd2*	TACCTGGACCGTTTCTTG	TGAGGCTTGATGGAGTTG
*h Ccnd3*	CTGGATCGCTACCTGTCTT	CCCACTTGAGCTTCCCTA
*h Ccne1*	GCCTTGTATCATTTCTCGTC	GCTGTCTCTGTGGGTCTG
*h Ccne2*	ACAATCATCTCCTGGCTAAA	GAGGTAAAATGGCACAAGG
*h Ccna2*	CAATGGATGGTAGTTTTGAGT	GTGATGTCTGGCTGTTTCTT
*h Cdk2*	AAGTTGACGGGAGAGGTG	AATGCCAGTGAGAGCAGA
*h P21*	GGGACAGCAGAGGAAGAC	GCGTTTGGAGTGGTAGAA
*h β-Actin*	GACATCCGCAAAGACCTG	GGAAGGTGGACAGCGAG
*m Ccna2*	GCCTTCACCATTCATGTGGAT	TTGCTGCGGGTAAAGAGACAG
*m Ccnd1*	GCGTACCCTGACACCAATCTC	CTCCTCTTCGCACTTCTGCTC
*m Ccnd2*	GAGTGGGAACTGGTAGTGTTG	CGCACAGAGCGATGAAGGT
*m Cdk4*	ATGGCTGCCACTCGATATGAA	TCCTCCATTAGGAACTCTCACAC
*m Ccne1*	GTGGCTCCGACCTTTCAGTC	CACAGTCTTGTCAATCTTGGCA
*m Ccne2*	ATGTCAAGACGCAGCCGTTTA	GCTGATTCCTCCAGACAGTACA
*m β-Actin*	CAGCCTTCCTTCTTGGGTAT	TGGCATAGAGGTCT TACGG
